# Targeting GD2 after allogeneic SCT: effector cell composition defines the optimal use of ch14.18 and the bispecific antibody construct NG-CU (GD2-CD3)

**DOI:** 10.1007/s00262-023-03536-x

**Published:** 2023-09-24

**Authors:** A.-M. Arendt, F. Heubach, C. P. Maier, S. Giardino, G. Jung, E. Kowalewski, A. Rabsteyn, G. Amorelli, C. Seitz, P. Schlegel, R. Handgretinger, P. Lang

**Affiliations:** 1 Department of General Pediatrics, Oncology/Hematology, Children’s University Hospital Tuebingen, Hoppe-Seyler-Str. 1, 72076 Tübingen, Germany; 2 Department of Hematology/Oncology, Center for Internal Medicine, University Hospital, Tübingen, Germany; 3 Interfaculty Institute for Cell Biology, Department of Immunology, Eberhard Karls University Tuebingen, Tübingen, Germany; 4 Hematopoietic Stem Cell Transplantation Unit, Department of Hematology and Oncology, IRCCS Istituto Giannina Gaslini, Genoa, Italy

**Keywords:** Antibody-based therapies, Stem cell transplantation, Neuroblastoma, GD2 antibodies

## Abstract

We investigated whether T cell-recruiting bispecific anti-CD3/GD2 antibody NG-CU might be an alternative to therapeutic anti-GD2 monoclonal antibody (mAb) ch14.18, mediating complement-dependent cytotoxicity (CDC) and antibody-dependent cell-mediated cytotoxicity (ADCC) through natural killer (NK) cells for immunotherapy in high-risk/relapsed neuroblastoma after autologous/allogeneic stem cell transplantation (auto/alloSCT). Different antibody concentrations and effector-to-target ratios (*E:T*) were evaluated using xCELLigence RTCA system, peripheral blood mononuclear cells (PBMCs) (healthy donors and patients after alloSCT), and neuroblastoma cell lines (LS/LAN-1). Mean specific lysis of LS cells utilizing PBMCs from healthy donors and ch14.18 (1 µg/ml) was 40/66/75% after 12/24/48 h compared to 66/93/100% in the presence of NG-CU (100 ng/ml). NG-CU showed enhanced cytotoxicity compared to ch14.18, even at lower concentrations and *E*:*T* ratios, and completely eradicated LS cells after 72 h. To decipher the influence of effector cell subsets on lysis, different ratios of T and NK cells were tested. At a ratio of 1:1, ch14.18 was more effective than NG-CU. Using patient PBMCs taken at different time points posttransplant, significant lysis with both constructs was detectable depending on percentages and total numbers of T and NK cells; in the early posttransplant phase, NK cells were predominant and ch14.18 was superior, whereas later on, T cells represented the majority of immune cells and NG-CU was more effective. Our study highlights the importance of analyzing effector cell subsets in patients before initiating antibody-based therapy. Consequently, we propose an adjusted administration of both antibody constructs, considering the state of posttransplant immune recovery, to optimize anti-tumor activity.

## Introduction

Neuroblastoma (NB) is the most common childhood extracranial solid tumor [1]. Its prognosis depends on the stage, patient age, molecular characteristics, and response to standard therapy. High-risk neuroblastoma (HR-NB) and especially refractory or relapsed (R/R) disease still represent a therapeutic challenge, displaying poor cure rates with standard protocols, reporting a 3- and 5-year event-free survival (EFS) of 6–50% [2–6]. The established therapeutic approach for HR-NB consists of multimodal approaches including surgical resection of the primary tumor, induction chemotherapy, high-dose chemotherapy (HD-CT) followed by autologous stem cell transplantation (autoSCT), and radiotherapy as maintenance therapy.

Targeted immunotherapy with monoclonal antibodies (mAbs) has shown significant results in the treatment of patients [7–9]. Anti-GD2 mAbs target the tumor-associated antigen (TAA) disialoganglioside GD2 on neuroblastoma cells, mediating ADCC via the Fc domain, which is mainly recognized by natural killer (NK) cells [10–12]. Moreover, these mAbs can activate the classical complement system pathway to elicit complement-dependent cytotoxicity (CDC).

GD2 is also expressed in healthy human tissues, but is primarily limited to neurons, peripheral sensory nerve fibers and skin melanocytes [13, 14]. This requires adequate multidrug analgesia to prevent neuropathic pain - the main side effect [15–17].

To date, a variety of anti-GD2 mAbs have been investigated in the clinical setting, and ch14.18/CHO (dinutuximab beta) [18, 19], a human/mouse chimeric mAb, has been approved for the treatment of HR-NB [20] and is currently used as first-line treatment after HD-CT and autoSCT.

In the haploidentical allogeneic SCT (alloSCT) setting, ch14.18 is used to take advantage of more potent donor-derived NK cells [18, 21, 22], which are predominant in the early posttransplant period while T cells recover only after a delayed period of time [22–26]. Thus, efficacy of mAb-mediated immunotherapy targeting GD2 could be affected by the immunological effector cell composition, which changes during posttransplant immune reconstitution toward a T cell predominance.

New bispecific antibodies (bsAbs) that instead recruit T cells are under investigation [18]. Lacking the Fc domain, bsAbs are not able to trigger ADCC/CDC but simultaneously bind TAAs on the surface of target cells and CD3 on T cells, resulting in activation and proliferation and ultimately in target cell elimination [27]. T cell activation through bsAbs is polyclonal, independent of major histocompatibility complex (MHC), T cell receptor (TCR) specificity, and co-stimulation, and hence can overcome escape routes exploited by tumors during the classic cytotoxicity of T cells [28].

Several clinical trials have demonstrated the impressive efficacy of bsAbs, e.g., blinatumomab, a CD19-CD3 bispecific T cell engager, which has been approved for the treatment of acute lymphoblastic B cell leukemia (B-ALL) in adults and pediatric patients [29, 30]. This success resulted in the development of a variety of GD2-CD3 mAb fusions and conjugates [31–33].

The bsAb 3F8BiAb, produced by chemical heteroconjugation of anti-CD3 (OKT3) and anti-GD2 (3F8), has been shown to redirect 3F8BiAb-armed T cells to specifically kill neuroblastoma cell lines in a non-MHC-restricted manner [31]. This approach has been further investigated in a phase I/II clinical trial, in which T cells were first armed with a GD2-CD3 bsAb (OKT3 X hu3F8) and subsequently infused back into patients with R/R NB [34].

In another approach, bsAbs were generated as tandem single chain variable fragments (scFvs) composed of two covalently linked scFvs, derived from affinity-matured anti-GD2 5F11 and the humanized anti-CD3 OKT3. A series of such antibody constructs has been characterized, and the most promising one has been proven to inhibit neuroblastoma xenograft growth [35]. The GD2-targeting hu3F8-BsAb, a tetravalent IgG-scFv, consists of the humanized anti-CD3 huOKT3 scFv, which is linked to the C-terminus of the humanized anti-GD2 hu3F8 IgG1 light chain, which is modified to prevent complement activation and cytokine storms. Hu3F8-BsAb was the first humanized anti-GD2 bsAb to demonstrate excellent anti-tumor activity both in vitro and in an in vivo neuroblastoma xenograft model and has been shown to penetrate solid tumors [32].

In contrast to the above-mentioned constructs, the bsAb NG-CU used in our study was designed as a fragment antigen-binding (Fab)-based construct (Fabsc), consisting of the Fab fragment of the murine anti-GD2 mAb 14.18 and the T cell-recruiting domain derived from the scFv of the anti-CD3 mAb UCHT-1. Both domains are linked by a CH2 domain, which is modified to attenuate FcR-binding. The Fabsc format more closely resembles the physiological structure of an antibody molecule and has been found to be superior to bsAbs with identical specificities regarding affinity to the target antigen, better production rates, and markedly lower aggregation tendencies [36]. The Fabsc format has previously been described for PSMAxCD3 and FLT3xCD3 and was developed by G. Jung and produced in a university-owned production unit [36, 37].

Here, we evaluated the cytotoxic activity of NG-CU in comparison to ch14.18 in the presence of different effector cells. Given the different designs of these two antibodies, we speculated that ch14.18 acts more efficiently in the presence of NK cells, whereas NG-CU activity relies predominantly on T cells. We show that NG-CU exceeded ch14.18-mediated lysis of neuroblastoma cell lines above a T:NK cell ratio of 1 - therefore, the state of posttransplant immune recovery should be considered for therapeutic antibody selection.

## Materials and methods

### Cell lines

LAN-1 (ACC 655) and LS (ACC 675) (neuroblastoma) were validated by STR-DNA-profiling or purchased from DSMZ and cultured in RPMI 1640 supplemented with 10% fetal calf serum, 100 U/ml penicillin, 100 µg/ml streptomycin, and 2 mM L-glutamine (all reagents Biochrom). Cells were tested for mycoplasma contamination before use.

### Patients

PBMCs were obtained from eight patients at various stages after alloSCT (range: 42–266 days posttransplant). Diagnoses included neuroblastoma relapse, neuroblastoma stage IV, atypical ganglioneuroblastoma, Krabbe disease, acute myeloid leukemia (AML), ALL, pro-B-ALL, and myelodysplastic syndrome (MDS). Five patients received T and B cell-depleted peripheral stem cells from a full haplotype-mismatched family donor (haploidentical donors), and three patients received unmanipulated grafts from matched unrelated donors (MUD).

### Ethics

The study was approved by the local ethics committee in accordance with the Declaration of Helsinki and written informed consent to participate and publish generated data was obtained from all individuals or legal guardians.

### Monospecific antibody

Monospecific GD2 antibody ch14.18 (dinutuximab) was obtained from APEIRON Biologics AG (Vienna, Austria).

### Bispecific antibody

**Fig. 1:**
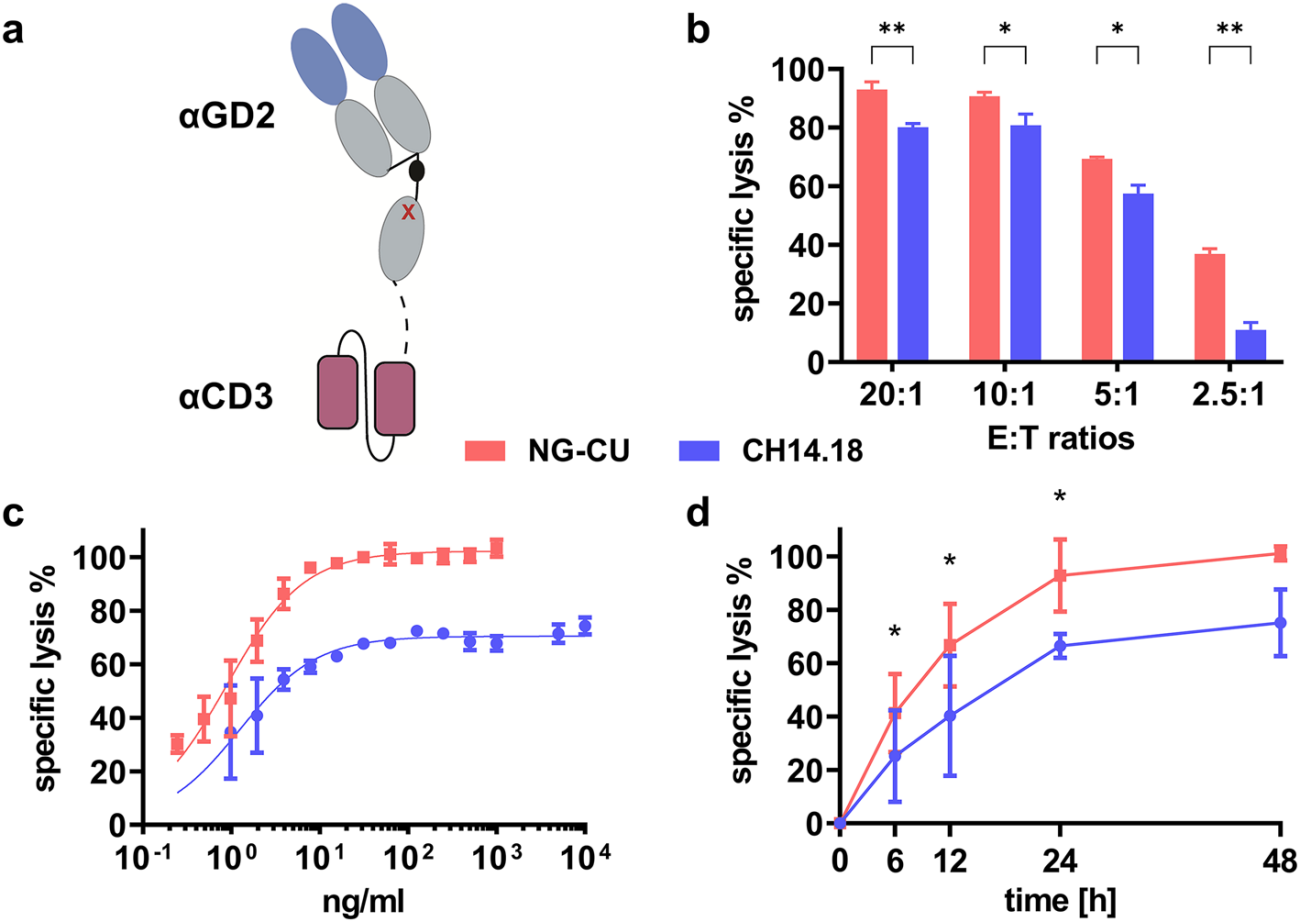
Schematic illustration of NG-CU and *E*:*T* and antibody titration of ch14.18 and NG-CU. **a** Schematic view of NG-CU. NG-CU is a recombinant (Fab)-based construct (Fabsc). It consists of a GD2-specific Fab fragment of the murine 14.18 antibody (blue) and a CD3-specific domain derived from the scFv of UCHT-1 (red). Both domains are linked by a CH2 domain containing an amino acid substitution (red x) to avoid FcR binding and a cysteine substitution (black dot) in the hinge region to prevent dimerization. **b** To determine the optimal *E*:*T* ratio, LS cells were incubated for 12 h with ch14.18 (1 µg/ml) or NG-CU (100 ng/ml), in the presence of PBMCs from 3 healthy donors (*E*:*T* ratios 20:1, 10:1, 5:1, 2.5:1). Cytotoxicity was assessed with the xCELLigence RTCA and specific lysis calculated as described. Shown are means + SD (*n =* 3). **c** Antibodies were titrated from 0.1 ng/ml to 10 µg/ml (NG-CU 0.1 ng/ml to 1 µg/ml; ch14.18 1 ng/ml to 10 µg/ml). Specific lysis of LS cells was determined after 24 h with PBMCs from 3 healthy donors as effector cells utilizing the xCELLigence assay. **d** Specific lysis of LS cells over time. LS cells were incubated with NG-CU (100 ng/ml), or ch14.18 (1 µg/ml), in the presence of PBMCs from healthy donors (*n =* 3, *E*:*T* = 5:1). Cytotoxicity was assayed using the xCELLigence RTCA and specific lysis calculated after 6, 12, 24, and 48 h. Shown are means + SD (*n =* 3)

NG-CU is a bsAb in recombinant Fabsc format. It was designed using a Fab fragment of the murine anti-GD2 14.18 antibody and the T cell-recruiting antibody domain derived from a scFv of the CD3-specific UCHT-1 antibody. Both domains were linked by a CH2 domain, which was modified to avoid FcR binding. Figure 1a represents a schematic illustration of this construct.

#### NG-CU heavy chain (mu14.18-UCHT-1)

EVQLLQSGPELEKPGASVMISCKASGSSFTGYNMNWVRQNIGKSLEWIGAIDPYYGGTSYNQKFKGRATLTVDKSSSTAYMHLKSLTSEDSAVYYCVSGMEYWGQGTSVTVSSASTKGPSVFPLAPSSKSTSGGTAALGCLVKDYFPEPVTVSWNSGALTSGVHTFPAVLQSSGLYSLSSVVTVPSSSLGTQTYICNVNHKPSNTKVDKKVEPKSCDKTHTSPPSPAPPVAGPSVFLFPPKPKDTLMISRTPEVTCVVVGVSHEDPEVKFNWYVDGVEVHNAKTKPREEQYQSTYRVVSVLTVLHQDWLNGKEYKCKVSNKQLPSPIEKTISKAKGQPSGDIQMTQSPSSLSASVGDRVTITCRASQDIRNYLNWYQQKPGKAPKLLIYYTSRLESGVPSRFSGSGSGTDYTLTISSLQPEDFATYYCQQGNTLPWTFGQGTKVEIKGGGGSGGGGSGGGGSEVQLVESGGGLVQPGGSLRLSCAASGYSFTGYTMNWVRQAPGKGLEWVALINPYKGVSTYNQKFKDRFTISVDKSKNTAYLQMNSLRAEDTAVYYCARSGYYGDSDWYFDVWGQGTLVTVSS.

#### NG-CU light chain (mu14.18-kappa)

DVVMTQTPLSLPVSLGDQASISCRSSQSLVHRNGNTYLHWYLQKPGQSPKLLIHKVSNRFSGVPDRFSGSGSGTDFTLKISRVEAEDLGVYFCSQSTHVPPLTFGAGTKLELKRTVAAPSVFIFPPSDEQLKSGTASVVCLLNNFYPREAKVQWKVDNALQSGNSQESVTEQDSKDSTYSLSSTLTLSKADYEKHKVYACEVTHQGLSSPVTKSFNRGEC.

### Density gradient centrifugation

PBMCs from healthy donors and patients were isolated from whole blood by density gradient centrifugation. Blood was mixed 1:2 with phosphate-buffered saline (PBS) and layered over a Biocol separation solution (Biochrom). After centrifugation lymphocytes were collected from the lymphocyte layer and washed three times with PBS.

### Flow cytometry (FACS)

GD2 expression on neuroblastoma cells was confirmed by FACS analysis using PE-labeled mouse anti-human antibody 14G2a (BioLegend). Stained cells were processed with a FACSCanto II instrument (BD Biosciences).

### xCELLigence real-time cytotoxicity assay (RTCA)

The xCELLigence® RTCA (ACEA Biosciences) system provides real-time, label-free and non-invasive monitoring of cell health by electrical impedance. This system uses microplates coated with gold electrodes (E-plates) on the bottom of each well. The impedance of current flow in each single well is measured as unitless parameter called cell index (CI) and depends on size, shape and number of adherent cells attaching to the surface of each well bottom. Upon cell death, CI decreases as a result of rounding and detaching of cells from the surface. Neuroblastoma cells were cultivated in E-plates (15.000 cells/well). After 48 h, target cells were incubated with effector cells and antibodies for up to 72 h. For calculations CI was normalized to the time of effector cell addition (NCI). Specific lysis was calculated according to the formula: ((NCI targets only-NCI test)/NCI targets only) × 100%.

### Statistics

The t test was used to compare two groups. P-values < 0.05 were considered significant.

### Software

GraphPad Prism 9.4.1 was used for statistical analysis (GraphPad Software LLC, Boston/MA, USA). FACS data were analyzed using FlowJo 10.8.0 (FlowJo LLC, Ashland/OR, USA). xCELLigence RTCA data were analyzed using RTCA Software 1.2 (ACEA Biosciences).

## Results

### Determination of the optimal effector target ratio

To determine the specific lysis mediated by ch14.18 or NG-CU, different *E*:*T* ratios (range: 20:1–2,5:1) were tested with PBMCs from healthy donors.

The highest lytic activity was detected using an *E*:*T* ratio of 20:1, nearly reaching 100% (80%) in the presence of NG-CU (ch14.18) using PBMCs as effector cells.

Lowering the *E*:*T* ratio reduced the specific lysis of LS cells in the presence of both antibody constructs. At *E*:*T* = 5:1, specific lysis, however, still ranged between 50 and 60%. Thus, we decided to utilize this *E*:*T* ratio (5:1) in further experiments, particularly as further *E*:*T* reduction substantially lowered specific lysis mediated by ch14.18 (Fig. 1b).

### Determination of saturating antibody concentrations

To find the optimum concentration for binding saturation, antibodies were titrated from 0.1 ng/ml to 10 µg/ml (NG-CU 0.1 ng/ml to 1 µg/ml; ch14.18 1 ng/ml to 10 µg/ml). LS cells were co-incubated with antibody constructs and PBMCs from 3 healthy donors (*E*:*T* = 5:1). Specific lysis was determined up to 48 h using the xCELLigence RTCA. After 24 h, target cell lysis by NG-CU nearly reached 100% even at a concentration as low as 100 ng/ml, whereas lysis by ch14.18 started plateauing at approximately 60% at the same concentration (Fig. 1c). Therefore, we chose saturating concentrations of 100 ng/ml for NG-CU and 1 µg/ml for ch14.18 for further experiments.

### Cytotoxic activity of ch14.18 and NG-CU in the presence of PBMCs

First, we analyzed the cytotoxic activity of ch14.18 (1 µg/ml) and NG-CU (100 ng/ml) against LS cells using PBMCs from three healthy donors (*E*:*T* = 5:1). Both antibody constructs showed increasing lytic activity over time; mean specific lysis after 12/24 h was ~ 40/66% (ch14.18) and ~ 66/93% (NG-CU). After 48 h, NG-CU showed specific target cell lysis of ~ 100%, whereas lysis by ch14.18 was limited to ~ 75% (Fig. 1d).

Thus, a higher specific lysis was mediated by NG-CU, especially after longer incubation times.

### Influence of the composition of effector cells on ADCC

**Fig. 2:**
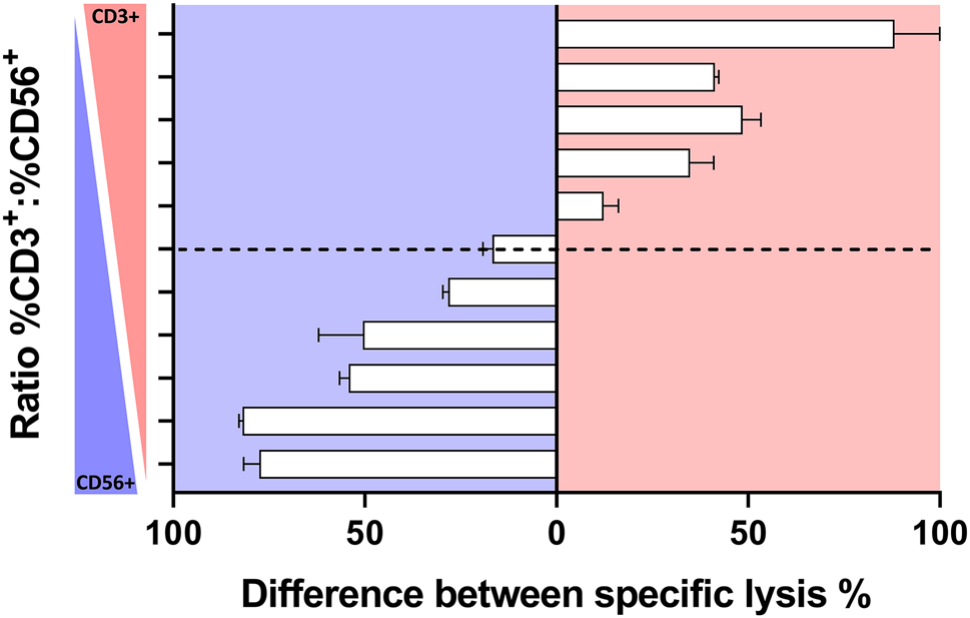
Difference between specific lysis of ch14.18 and NG-CU. CD3^+^ and CD56^+^ cells were isolated from PBMCs of healthy donors and mixed in ratios from 0% NK cells (100% T cells) to 100% NK cells (0% T cells). Subsequently, specific lysis against LS (*E*:*T* = 5:1) was measured with both antibodies separately for each ratio. The y-axis shows the ratio of %CD3 to %CD56, the dotted line indicates a mixture of T and NK cells of 1:1. The x-axis shows the difference of specific lysis measured for both antibodies separately at each respective ratio. Difference was calculated as %spec. lysis NG-CU-%spec. lysis ch14.18. Depending on which antibody mediated the stronger lytic effects of target cells, bars point either to the right (NG-CU = red) or to the left (ch14.18 = blue). Shown are means + SD (*n =* 3)

Next, we evaluated the role of effector cell subsets in ADCC. CD3^+^ T cells and CD56^+^ NK cells were isolated using magnetic microbeads from PBMCs of healthy donors. Purified NK cells were then mixed with purified T cells in percentages from 0% NK cells (100% T cells) to 100% NK cells (0% T cells). Subsequently, for each ratio, target cell lysis was monitored with both antibodies separately (*E*:*T* = 5:1). Figure 2 shows the difference between specific lysis of NG-CU and ch14.18 (calculated as %spec. lysis NG-CU-%spec. lysis ch14.18), which reflects the dominant antibody/effector cell combination at the respective ratios. With decreasing NK cell counts the difference between antibody-induced lysis is reduced, indicating NG-CU-mediated target cell lysis gradually increases while the opposite is true for ch14.18; however, even at a T:NK ratio of 1:1, antibody-mediated lysis still is in favor for ch14.18 (Fig. 2, blue background). By further increasing the T cell count above the ratio of 1:1, the difference of specific lysis starts to increase again, but this time in favor for NG-CU-mediated killing (Fig. 2, red background). Taken together, target cell lysis is strongly dependent on the composition of effector cell populations.

### Influence of complement-dependent cytotoxicity on target cell lysis

To investigate whether both antibody constructs not only mediate ADCC but also CDC, specific lysis of LS cells was additionally measured in the presence of autologous serum. Therefore, LS cells were incubated with ch14.18, NG-CU, PBMCs from healthy donors and/or normal or heat-inactivated autologous serum. To pronounce the cytolytic effect mediated by CDC in this assay, *E*:*T* was adjusted to 1:1.

**Fig. 3:**
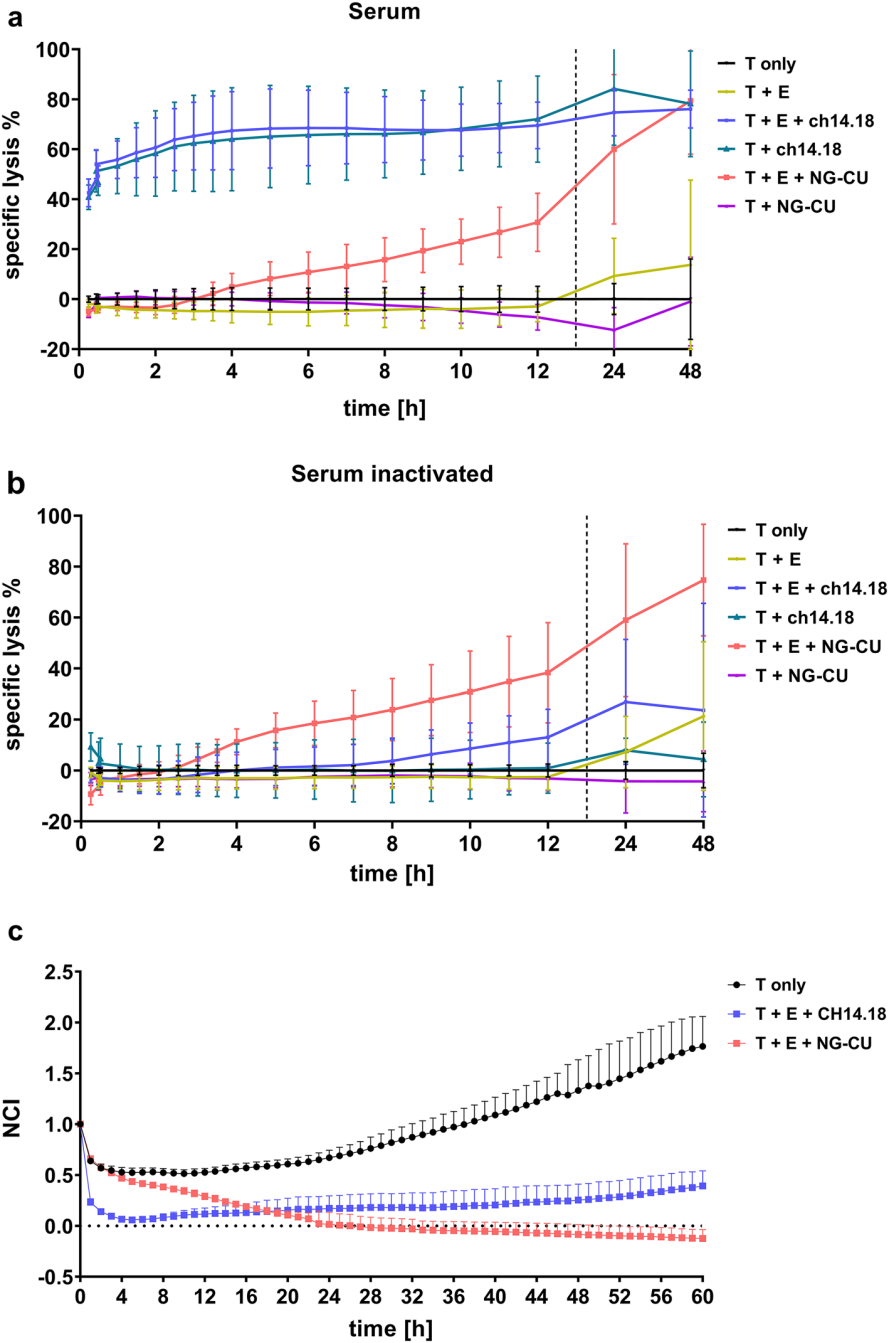
Influence of complement on ch14.18/NG-CU-mediated killing. **a** Specific lysis of LS cells incubated with or without PBMCs (1:1) from healthy donors (*n =* 3), ch14.18 (1 µg/ml), NG-CU (100 ng/ml) and autologous serum that has not been heat-inactivated. **b** Specific lysis of LS cells incubated under the same conditions but with heat-inactivated autologous serum. **c** NCI of LS cells incubated with PBMCs (1:1) from healthy donors (*n =* 3), ch14.18 (1 µg/ml), NG-CU (100 ng/ml) and autologous serum. **a**–**c** Specific lysis/NCI as determined by xCELLigence RTCA assays. Shown are means + SD (*n =* 3)

Cytotoxicity of ch14.18 was drastically increased in the presence of autologous serum and reached 67% and 80% after 4 h and 48 h, respectively (Fig. 3a). Interestingly, overall lysis by ch14.18 was not reduced in the absence of effector cells. In contrast, using heat-inactivated serum, lysis by ch14.18 was tremendously diminished, despite the presence of PBMCs. Utilizing NG-CU instead of ch14.18, no lysis at all was detected in the absence of PBMCs, irrespective of the presence or absence of functional serum (Fig. 3b). Altogether, these results indicate that the lytic effect of ch14.18 against tumor cells also depends on the presence of functional complement, whereas overall lysis by NG-CU could not be further enhanced by adding complement. Looking at the growth of LS cells during the ongoing assay to further analyze the impact of CDC in the presence of ch14.18 and low effector cell counts revealed, that target cells were indeed efficiently killed within hours but not completely eradicated. Instead LS cells showed steady cell growth despite continual antibody treatment with ch14.18 (Fig. 3c). As a result, ch14.18 is not capable of eradicating tumor cells exclusively by CDC, but also requires a minimum number of effector cells to additionally display efficient ADCC. In contrast, NG-CU managed to eradicate LS cells completely after 48 h of treatment.

### Cytotoxic activity of ch14.18 and NG-CU in the presence of PBMCs from patients after SCT

**Fig. 4:**
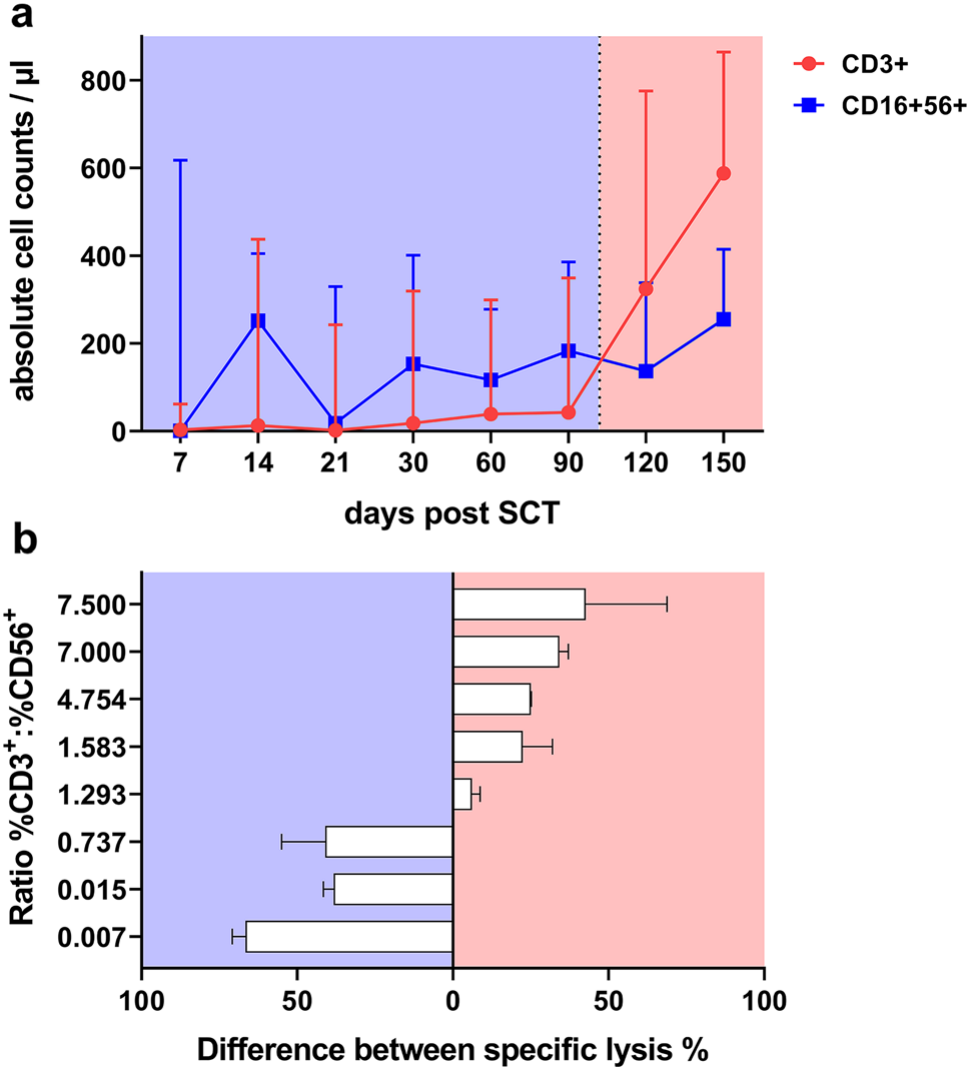
Influence of PBMC composition collected from patients after SCT on the cytotoxicity of ch14.18 and NG-CU. **a** Immune reconstitution of T and NK cells of 8 patients after SCT. In the first months of immune reconstitution NK cells represented the majority of effector cells, between day 90 and day 120 T cell counts exceeded NK cell counts (means + SD). **b** Difference between specific lysis induced by ch14.18 and NG-CU and patient PBMCs collected at different time points after SCT. Specific lysis against LS or LAN-1 (*E*:*T* = 5:1) was measured with both antibodies separately for each patient. The y-axis shows the ratio of %CD3 to %CD56. The x-axis shows the difference of specific lysis measured for both antibodies at the respective ratio, i.e., for each patient. Difference was calculated as %spec. lysis NG-CU-%spec. lysis ch14.18. Depending on which antibody mediated the stronger lytic effects on target cells, bars point either to the right (NG-CU = red) or to the left (ch14.18 = blue). Shown are means + SD (*n =* 3 technical replicates)

In addition, we analyzed the cellular composition (T:NK ratio) and cytolytic activity of effector cells obtained from patients at different stages after alloSCT (haplo donor (*n =* 5); MUD (*n =* 3)). CD56^+^ NK cells represented the dominant effector cell population in patients early after SCT, whereas CD3^+^ T cell recovery significantly improved on day + 120 (Fig. 4a).

Consequently, and in line with the results depicted in Fig. 2, considerable amount of target cell lysis with both constructs was detected and the cytolytic potential of patient effector cell populations varied depending on the T:NK ratio and the antibody used (Fig. 4b). Above a T:NK cell ratio of around 1 bispecific NG-CU induces stronger lysis than ch14.18. With PBMCs taken early after SCT, ch14.18 elicited higher target cell lysis, whereas using effector cells collected later on and thus containing increasing amounts of T cells, NG-CU was more effective.

**Fig. 5:**
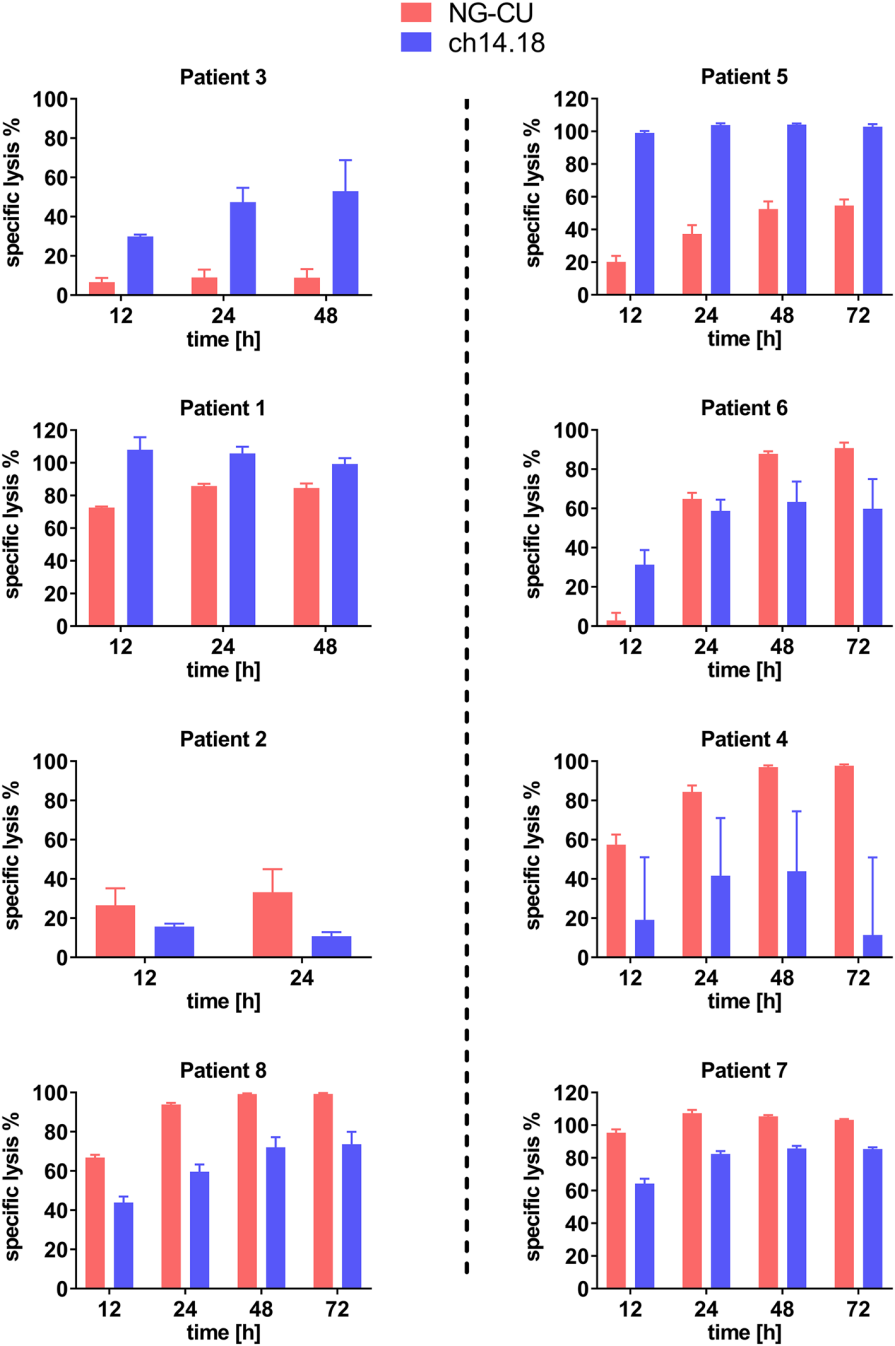
NG-CU and ch14.18-mediated cytotoxicity using patient PBMCs. Cytotoxicity mediated by both antibody constructs in combination with patients’ PBMCs collected at different time points after SCT, i.e., containing a different effector cell composition, was assessed for up to 72 h using the xCELLigence RTCA system. Neuroblastoma target cells: LS (left panel), LAN-1 (right panel). In total, we evaluated effector cells from 8 patients separately. From top to bottom T cell content within the respective effector cell population increases. Shown is the calculated specific lysis (%) after different incubation times (mean + SD of 3 technical replicates per patient)

Figure 5 shows target cell lysis of neuroblastoma cell lines LS and LAN-1 with PBMCs collected from the same patients. Both antibodies mediate target cell lysis in concert with patients’ PBMCs. Depending on the amount of T cells present within the respective PBMC population, target cell lysis mediated by NG-CU varies considerably. From top to bottom of Fig. 5 T cell counts increase, which is reflected by the increase of cytotoxicity mediated by NG-CU.

**Fig. 6:**
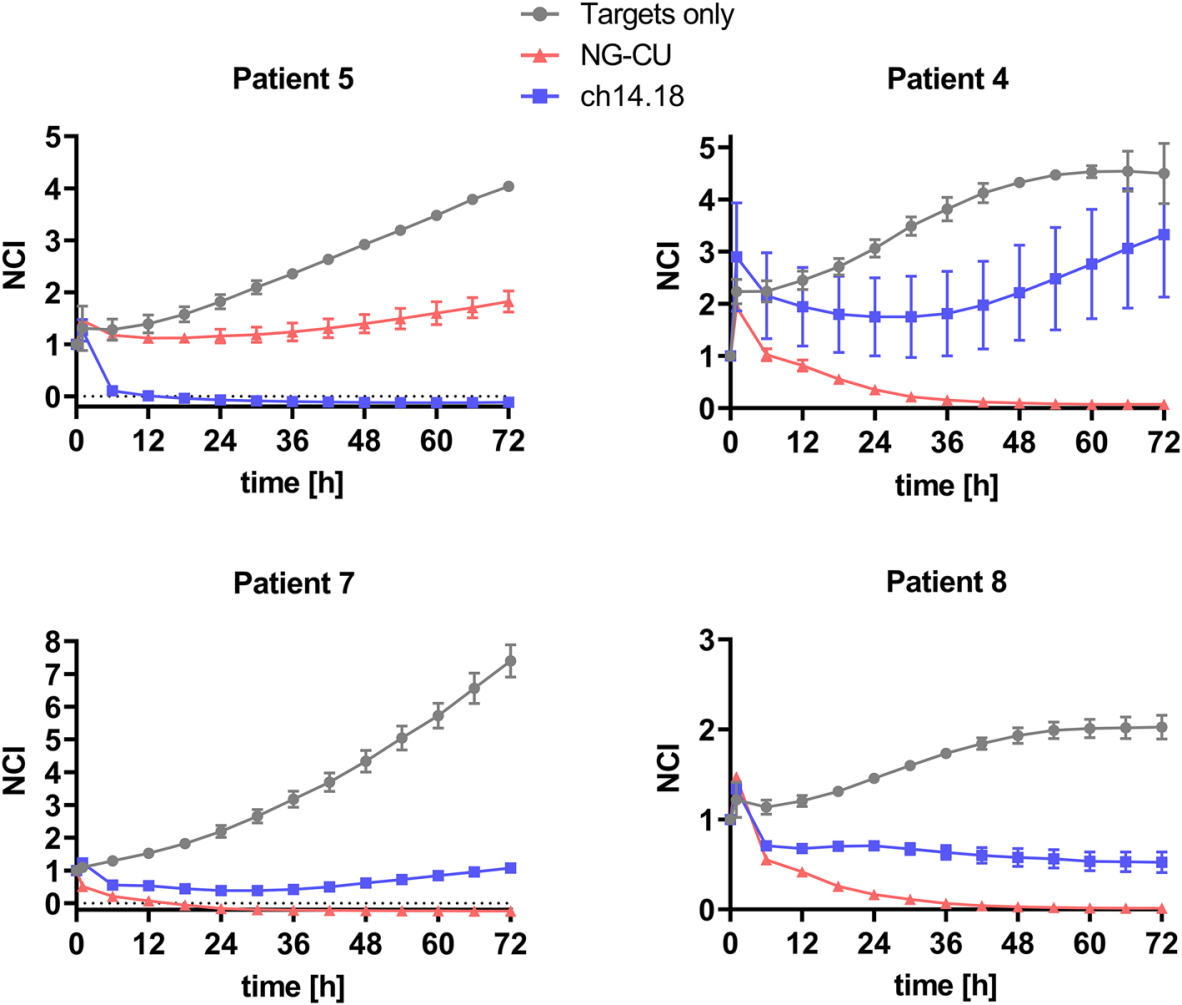
Kinetics of NG-CU and ch14.18-mediated tumor cell lysis in combination with patient PBMCs. LS or LAN-1 target cells were incubated with patient PBMCs and NG-CU (100 ng/ml) or ch14.18 (1 µg/ml). Cell growth and thus target cell lysis was assessed using the xCELLigence RTCA system for up to 72 h. Shown is the NCI as a measure for cell growth of target cells alone (gray) and of target cells incubated with effector PBMCs and antibodies (NG-CU = red, ch14.18 = blue), normalized to the time of PBMC and antibody addition (mean + SD of 3 technical replicates per patient)

Looking at four exemplary killing kinetics in detail (Fig. 6), PBMCs of patient 5 completely eradicated target cells in combination with ch14.18, whereas NG-CU mediated only partial lysis, which consequently resulted in slow but steady tumor cell growth over time. This can be explained by the low number of T cells and the relative high amount of NK cells (i.e., low T:NK ratio) within this patient’s PBMCs. In contrast, patients with a higher percentage of T cells (patients 4, 7, 8) showed profound and lasting target cell lysis in combination with NG-CU, whereas ch14.18-mediated lysis was not sufficient to kill all tumor cells.

### Effect of combined antibody use on cell lysis

**Fig. 7:**
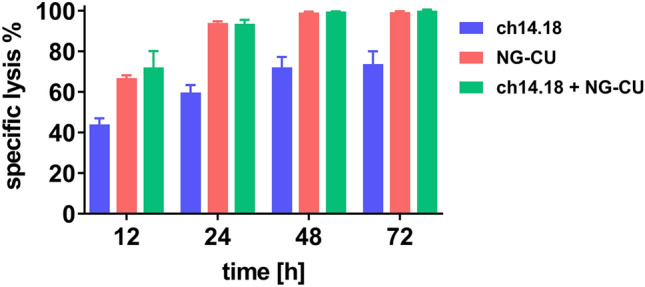
Combined application of both antibody constructs, compared to single use. Lysis of LS cells by PBMCs in combination with one or both antibody constructs was assessed after 12, 24, 48, and 72 h using the xCELLigence RTCA assay. PBMCs from a patient collected > 200 days after alloSCT were used as effector cells (*E*:*T* = 5:1). Shown are means + SD (*n =* 3 technical replicates)

The combined use of both antibody constructs resulted in only a small additive effect. (Fig. 7). Especially after a longer incubation time (≥ 24 h), the combination of both constructs was not superior with regard to lysis compared to NG-CU alone. Thus, instead of combined treatment with both antibodies, a time-shifted treatment adapted to immune reconstitution seems to be the most promising treatment strategy.

## Discussion

In our study, we analyzed the cytotoxic function of NG-CU, a new GD2-targeting and T cell-recruiting Fabsc construct, and compared its efficacy on lysis with the classical monospecific ch14.18.

Currently, two anti-GD2 mAbs have been approved for clinical use in HR-NB children, hu3F8 (naxitamab) and ch14.18 (dinutuximab), the latter being used as standard first-line immunotherapy after HD-CT and SCT.

In the past decade, several clinical trials have demonstrated the clinical success of ch14.18 as an effective immunotherapeutic agent against neuroblastoma and to significantly improve survival of stage IV NB patients [7–9, 20, 38]. However, not all patients response to anti-GD2 treatment. Although survival rates increased in HR patients treated with anti-GD2 mAb in combination with cytokines, only 63% of stage IV patients remained free of disease at 2 years [7]. Survival rates in relapsed patients are much poorer. Thus, there is an ongoing clinical need for innovative therapeutics for HR-NB, especially in patients with relapse.

Therefore, we evaluated the use of allogeneic SCT with haploidentical donors using *ex vivo* T-/B cell-depleted grafts followed by immunotherapy with dinutuximab beta (ch14.18/CHO) in children with stage IV relapsed neuroblastoma [22]. The aim was to increase the graft-versus-NB effect by recruiting more effective donor-derived NK and γδ T cells mediating ADCC, overcoming the limitations related to autologous NK cells [39, 40].

The 5-year EFS was significantly improved by GD2 mAb infusions compared with a historical cohort of patients who received only HSCT (43% vs. 19%) [22, 39, 41]. Accompanying investigations have revealed tumor control by ADCC and CDC, highly functional NK cells, and clinically relevant serum levels of dinutuximab beta [21]. However, approximately 50% of patients showed either stable or progressive disease. Thus, we hypothesized that additional use of T cells during the posttransplant recovery phase, might be beneficial.

Using PBMCs from healthy donors as effector cells, we showed that ch14.18 and NG-CU significantly differed in their capacity to lyse neuroblastoma cells. Although the concentration of NG-CU (100 ng/ml) was reduced to one-tenth of that used for ch14.18 (1 µg/ml) in our in vitro assays, specific lysis by NG-CU remained superior, particularly at low *E*:*T* ratios. Additionally, lysis mediated by NG-CU occurred faster; after 12 h, NG-CU exceeded ch14.18-mediated mean lysis by 26%. Both antibodies reached maximum lytic activity at similar concentrations. However, NG-CU elicited > 90% specific lysis, whereas lysis by ch14.18 in the presence of PBMCs was restricted to 60% after 24 h. Given the fundamental differences in their structure and, therefore, in their mode of action, it seemed to be essential to evaluate both antibodies not only in the presence of PBMCs, but also with respect to subsets of PBMCs and complement. Analysis of antibody-mediated lysis depending on different T:NK ratios clearly revealed that an increasing T:NK ratio also resulted in increased NG-CU-mediated lysis. As assumed, since NG-CU lacks the Fc domain, lysis was not further enhanced by adding functional complement. On the contrary, ch14.18-mediated lysis was increased at lower T:NK ratios, and remarkably enhanced (by > 50% at 2 h, *E*:*T* = 1:1) in the presence of autologous sera, indicating that complement is a prerequisite for full functioning of ch14.18.

The different modes of action of ch14.18 and NG-CU are also clinically relevant: To reach an optimum anti-tumorous effectiveness, clinicians should be aware of patients’ effector cell composition before initiating antibody treatment. This issue is of great interest, especially in the alloSCT setting. As mentioned, haploSCT with manipulated T-/B cell-depleted grafts followed by GD2-based immunotherapy is currently being investigated with promising results [24, 37]. This transplantation approach often leads to delayed recovery of donor-derived T cells after transplantation at approximately day + 100, whereas donor-derived NK cells reconstitute in the first weeks and form an NK cell wave with high CD56^+^ counts starting at day + 14 [23–26, 42]. Moreover, a considerable proportion of patients who received SCT with grafts from HLA-matched donors showed low T cell numbers within 3 months posttransplant [43]. Thus, we can speculate that mAb-mediated immunotherapy targeting GD2 could be affected by the efficiency of immunological effector cell composition, which changes during posttransplant immune reconstitution.

We addressed this issue in vitro by using PBMCs of patients as effector cells at various time points after alloSCT, (therefore) displaying a vast spectrum of T:NK ratios (range:0.007–7.5). In line with our results from assays using T and NK cells from healthy donors, our data revealed that ch14.18 was superior to NG-CU early after alloSCT at lower T:NK ratios, whereas NG-CU performed better after d + 100 in the presence of higher T cell counts. These results clearly reflect the dependence of both antibody constructs on their corresponding effector cells, which on their own quantitatively depend on the stage of posttransplant immune reconstitution.

Besides the influence of conditioning, graft manipulation, and T cell-depleting agents on recovery and functional properties of NK and T cells, immunotherapy itself may have a significant impact on effector cell functions. Recently, Philipp et al*.* [44] showed for the first time that the usual continuous 28-day application of blinatumomab, a T cell-recruiting bsAb (CD3xCD19) widely used in R/R B-ALL therapy, led to a progressive decline in T cell function, which was efficiently restored by introducing treatment-free intervals. Further, it is of great importance to decipher the underlying mechanisms of treatment failure. The composition of the tumor microenvironment (TME) might play a crucial role in this context and can impair immunotherapy in neuroblastoma in various ways [45]. Here, we describe the additional use of T cells recruited by the Fabsc NG-CU following ch14.18 and NK cell-mediated immunotherapy in neuroblastoma post alloSCT. Yet, this approach bears the risk of treatment failure upon antigen loss, since ch14.18 and NG-CU both target GD2. However, by using two different effector cell populations, it might be possible to compensate for certain immunosuppressive TME features that impair in particular one or the other effector cell type. Additionally, in the case of T cells, activation through bsAbs is independent of MHC, TCR specificity, and co-stimulation, and hence might overcome certain immunosuppressive effects of the TME and complement initial NK cell-mediated immunotherapy [28].

## Conclusion

In conclusion, our in vitro investigations showed that the high efficiency of bsAb NG-CU may represent an addition to classic ch14.18-mediated immunotherapy capable of enhancing the killing of NB cells by T cell recruitment. Translating these findings into a potential clinical application in HR-NB patients, we hypothesized that the sequential administration of both ch14.18 and NG-CU antibodies in relation to the state of immune reconstitution after allogeneic SCT (especially with haploidentical donors) could enhance the graft-versus-NB effect and become a more efficient alternative to classical approaches.

## Data Availability

All contributors had access to the primary data, approved the manuscript, and agreed with the data presented.
